# A randomized phase II trial of nab-paclitaxel with or without mifepristone for advanced triple-negative breast cancer

**DOI:** 10.1007/s10549-025-07626-5

**Published:** 2025-02-10

**Authors:** Nan Chen, Margarite Matossian, Poornima Saha, Murtuza Rampurwala, Salaija Kamaraju, Olwen Hahn, Frederick M. Howard, Gini F. Fleming, Jincong Q. Freeman, Theodore Karrison, Suzanne Conzen, Rita Nanda, Erica M. Stringer-Reasor

**Affiliations:** 1 Department of Medicine Section of Hematology/Oncology, University of Chicago, Chicago, IL, USA; 2 Department of Medicine, NorthShore University Health System, Evanston, IL, USA; 3 Department of Medicine, Medical College of Wisconsin, Milwaukee, WI, USA; 4 Department of Public Health, University of Chicago, Chicago, IL, USA; 5 Department of Biostatistics, University of Chicago, Chicago, IL, USA; 6 Department of Medicine Section of Hematology/Oncology, University of Texas Southwestern, Dallas, TX, USA; 7 Department of Medicine, University of Alabama at Birmingham, Birmingham, AL, USA

**Keywords:** Glucocorticoid receptor, HER2- breast cancer, Clinical trial, Mifepristone, Nab-paclitaxel

## Abstract

**Purpose:**

Glucocorticoid receptor (GR) activity may mediate chemoresistance in advanced triple-negative breast cancer (TNBC). Preclinical studies demonstrate that GR antagonism can augment the effect of taxanes in TNBC models. We hypothesized that pretreatment with mifepristone, a potent GR antagonist, would enhance nab-paclitaxel efficacy in advanced TNBC.

**Methods:**

This trial was terminated early due to poor accrual. 29 of 64 planned patients were enrolled. Patients were randomized to receive nab-paclitaxel with or without mifepristone; oral mifepristone 300 mg was administered the day prior and day of each dose of nab-paclitaxel. The primary endpoint was progression-free survival (PFS); secondary/exploratory endpoints included response rate and correlation of response with GR expression.

**Results:**

The addition of mifepristone to nab-paclitaxel did not improve PFS (3.0 m vs 3.0 m, *p* = 0.687) or overall response rate (23% vs 31.5%) compared to nab-paclitaxel alone. There was a trend towards improved overall survival in the combination group, primarily driven by one long-term responder. Increased rates of grade 3 neutropenia (46% vs 7%) and febrile neutropenia were observed in the combination arm, while other toxicities were similar in both groups. Increased GR expression was not correlated with clinical response in the combination arm.

**Conclusions:**

While there were responders to the combination, the study was underpowered to meet the primary endpoint. Higher rates of neutropenia were observed in the combination, but overall it was well tolerated. Preclinical data in TNBC and clinical data in other malignancies support further investigation of GR modulators. Future studies should incorporate biomarkers to select patients who benefit from GR inhibition.

## Introduction

Metastatic triple-negative breast cancer (mTNBC) is an aggressive disease with limited treatment options [1, 2]. While novel therapies have improved outcomes and targeted treatments have redefined the treatment landscape, chemotherapy remains the backbone of therapy and strategies to reduce chemotherapy resistance are needed [1-4]. Nab-paclitaxel is an albumin-bound, solvent-free formulation of the insoluble drug paclitaxel that reduces the need for pre-medications, including steroids [5]. A phase II study evaluating nab-paclitaxel 100 mg/m^2^ or 125 mg/m^2^ on days 1, 8, and 15 of a 28-day cycle demonstrated response rates of 14–16% in taxane-resistant, pretreated metastatic breast cancer patients [6]. Unfortunately, many tumors do not respond to nab-paclitaxel, and those that do eventually develop resistance. Innovative approaches to enhance tumor sensitivity to taxanes are needed.

The glucocorticoid receptor (GR) is expressed in significant subsets of TNBC [7]. High GR expression in primary TNBC is associated with a significantly increased probability of relapse [8]. Data in ER-negative cancer cell lines demonstrate that activation of GR drives mammary tumor growth [9] and initiates cell-survival pathways under otherwise apoptosis-inducing conditions such as chemotherapy [8, 10]. In preclinical models, GR activation inhibits taxane-induced apoptosis[11] and is associated with taxane resistance [12]. Mifepristone is a highly potent GR antagonist [13]. GR antagonism with mifepristone augmented the antitumor efficacy of taxanes in GR-positive TNBC xenografts [14]. We hypothesized that GR antagonism would improve responses to nab-paclitaxel by inhibiting cortisol-mediated cell-survival pathways that would otherwise counteract taxane-induced apoptosis in tumor cells.

Our group previously published a phase I trial of nab-paclitaxel with or without mifepristone that demonstrated a manageable safety profile of mifepristone 300 mg in combination with nab-paclitaxel at a dose of 100 mg/m^2^.[15] Neutropenia occurred in many patients at both nab-paclitaxel dose levels studied, but was mitigated with dose reduction and/or growth factor administration. Additionally, promising efficacy was observed in patients with GR-positive TNBC, with 4 of 6 patients having a response to therapy (2 complete responses [CR], 2 partial responses [PR]). Another recent phase I trial explored effects of the selective GR modulator relacorilant with nab-paclitaxel in solid tumors including breast cancer. [16] This yielded a 33% disease control rate at 16 weeks with 28.6% patients experiencing a longer duration of benefit than on their prior taxane regimen [16]. Findings from both phase I trials provided promising data to further explore GR antagonism in combination with nab-paclitaxel.

## Patients and methods

This was a randomized, double-blinded, placebo-controlled phase II study evaluating the safety and efficacy of nab-paclitaxel with or without mifepristone in patients with advanced TNBC (ClinicalTrials.gov identifier: NCT02788981). This study was conducted at the University of Chicago (Chicago, IL), Northshore University Health System (Evanston, IL), Medical College of Wisconsin (Milwaukee, WI), and the University of Alabama (Birmingham, AL). This study was approved by Institutional Review Boards at each respective institution. All participants provided written informed consent prior to study enrollment.

### Eligible patients and study treatment

Eligible patients were ≥ 18 years old, had locally advanced unresectable or mTNBC (defined as ER/PR < 10%, HER2-negative), RECIST measurable disease, and had not previously received nab-paclitaxel in the early or advanced disease settings. [2] Patients could have received up to 2 prior lines of chemotherapy in the metastatic setting. Patients with previously treated stable brain metastases were allowed to participate. Exclusion criteria included pre-existing peripheral neuropathy ≥ grade 2. Patients were randomized 1:1 to receive nab-paclitaxel 100 mg/m^2^ (in both arms) on days 1, 8, 15 of each 28-day cycle with either mifepristone 300 mg or placebo on the day prior to and day of each dose of nab-paclitaxel. If nab-paclitaxel was delayed or skipped, then mifepristone doses were also omitted. Dose reductions of nab-paclitaxel were allowed at investigator discretion. Dose reductions of mifepristone were not permitted, but it could be held or discontinued due to toxicity. Patients had radiologic evaluation of clinical response every 8 weeks after start of therapy; response was evaluated by RECIST 1.1 criteria. [17] Granulocyte colony-stimulating factor (G-CSF) was allowed during Cycle 1.

### Study objectives

The primary objective of the study was to compare the progression-free survival (PFS) in patients treated with nab-paclitaxel with placebo vs nab-paclitaxel with mifepristone. The secondary objectives were overall response rate (ORR) defined as achieving a PR or CR at any point during therapy, overall survival (OS), and correlation of GR positivity with PFS. Safety and tolerability were also evaluated. All adverse events (AEs) were recorded and monitored. Treatment-emergent AEs (TEAE) were graded using Common Terminology Criteria for Adverse Events. AEs were determined to be related or unrelated to study drugs by the treating investigator.

### Drug supply

Nab-paclitaxel was obtained commercially in single-dose vials (manufactured by Celgene). Mifepristone (300 mg tablets) and matching placebo tablets were supplied by Corcept Therapeutics.

### Determination of GR expression

If available, archival tissue was used to assess GR expression for each patient via IHC. An anti-rabbit monoclonal antibody against GR ([D8H2; Cell Signaling Technology 9#3660S] Danvers, MA, USA) was utilized. A percent score was used to semi-quantitatively assess tumor GR expression in samples with at least 100 viable invasive carcinoma cells. The intensity of nuclei staining was reported based on the H-score method using 0 for negative staining, 1 + for weak staining, 2 + for moderate staining, and 3 + for strong staining. For this assay, GR positivity was defined as ≥ 10% nuclear staining of tumor cells at any intensity. A board-certified pathologist scored nuclear tumor staining in the total area of viable tissue section available. All stained slides were reviewed by the pathologist using H-scores, which range from 0 to 300 and consist of the total sum of 1 × the percentage of cells with weak nuclear staining, 2 × the percentage of cells with moderate nuclear staining, and 3 × the percentage of cells with strong nuclear staining. The validation of this assay has been previously described. [18]

### Statistical analyses

This study utilizes a Simon’s two-stage minimax design and had a planned accrual of 64 patients (32 patients per arm) to provide 80% power (at one-sided alpha of 0.15) to detect a hazard ratio (HR) of 0.61, which corresponds to a median PFS of 5.2 months in the combination arm compared to an expected median PFS of 3.2 in the nab-paclitaxel monotherapy arm. In the first stage, 22 patients will be accrued. If there are 2 or fewer responses in the first 22 patients enrolled, the study will be discontinued for futility. Baseline characteristics and safety data were summarized by treatment arm in patients who received at least one dose of either study medication. OS and PFS were analyzed using the Kaplan–Meier method, followed by Cox proportional hazard regression. Median survival time (in months) and 95% confidence intervals (95% CI) were calculated. P values for H-score by response status were calculated using ANOVA or Kruskal–Wallis tests. All analyses were performed using Stata 17 (StataCorp, College Station, TX).

## Results

### Patient characteristics

A total of 29 patients were enrolled between September 2017 and July 2021. Baseline characteristics are summarized in Table 1. Sixteen patients were randomized to nab-paclitaxel with placebo, and thirteen patients to nab-paclitaxel with mifepristone. One patient withdrew from the study after randomization to the placebo arm to pursue alternative therapy prior to receiving any study treatment and 28 patients received at least one dose of study treatment. The mean age was 53 years (range 32–73) and 38% of patients self-identified as Black. 10% of patients had no prior lines of therapy, 21% patients had 1 prior line of therapy, 17% of patients had 2 prior lines of therapy; 52% patients were unknown. (Table 1).

### Clinical response

**Fig. 1:**
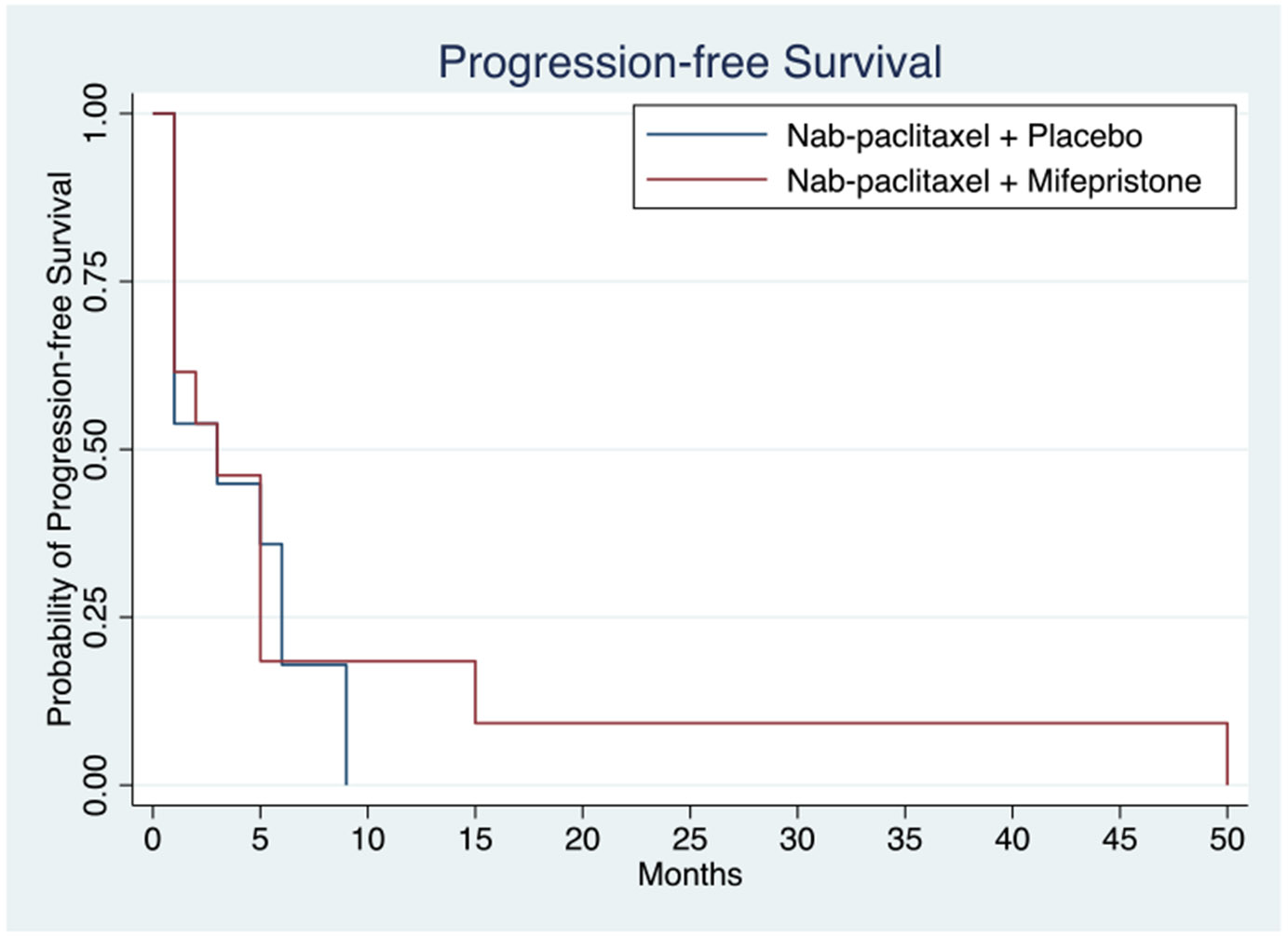
Progression-free survival in patients treated with nab-paclitaxel with or without mifepristone. Median progression-free survival in the nab-paclitaxel with placebo arm (*n* = 13) and nab-paclitaxel combined with mifepristone (*n* = 13) were 3 and 3 months, respectively, with *P* value 0.687

**Fig. 2:**
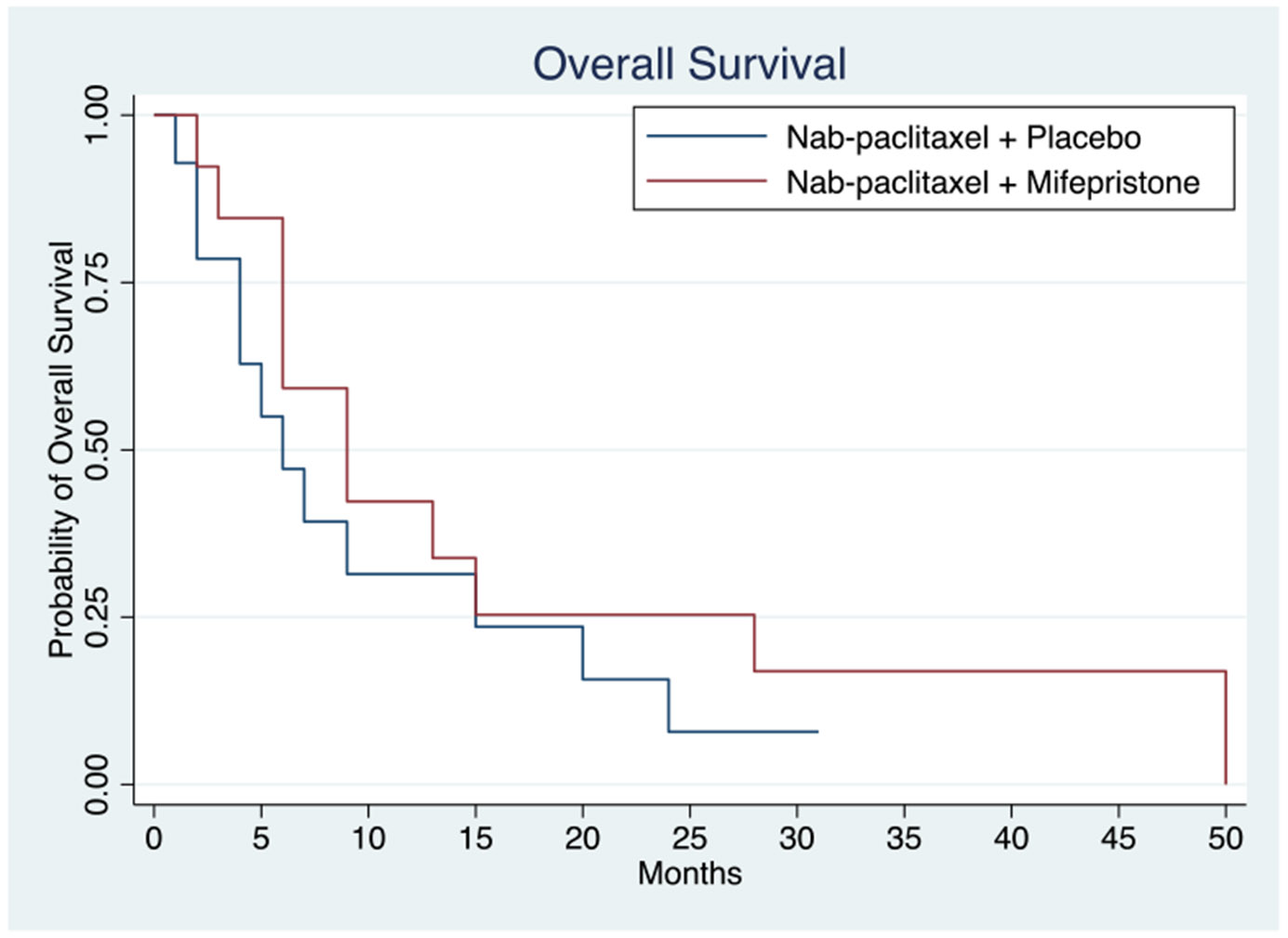
Overall survival in patients treated with nab-paclitaxel with or without mifepristone. Median overall survival in the nab-paclitaxel with placebo arm (*n* = 13) and nab-paclitaxel combined with mifepristone (*n* = 13) were 6 and 9 months, respectively, with *P* value 0.325

Clinical outcomes of patients treated with nab-paclitaxel with placebo or in combination with mifepristone are reported in Table 2. The median follow-up time was 7.5 months. Of the 29 patients enrolled in the study, 26 patients were evaluable for response. Two patients who received study treatment came off study prior to first imaging timepoint and did not have any radiographic assessment of clinical response; 1 patient withdrew to pursue alternative therapy, and 1 patient received less than one cycle of study therapy, experienced grade 3 febrile neutropenia, and was taken off study by the investigator. Kaplan–Meier curves for PFS by treatment arm are shown in Fig. 1. The median PFS was 3.0 and 3.0 months (mos) (HR = 0.87, 95% CI 0.37 – 2.01, *p* = 0.687) in the nab-paclitaxel group alone and combination arms, respectively (Table 2). ORR in the nab-paclitaxel and combination arms were 31.5% and 23%, respectively; both arms had 1 CR. Figure 2 shows Kaplan–Meier curves for OS of the two treatment arms. Median OS with nab-paclitaxel alone was 6.0mos and in the combination arm was 9.0mos. Compared to the placebo arm, the treatment arm had a lower mortality risk, though not statistically significant (HR = 0.67, 95% CI 0.29 – 1.16, *p* = 0.325) (Table 2). The patient on the mifepristone combination arm who achieved a CR completed 50 cycles and died of a non-cancer, non-treatment-related event. One patient on the nab-paclitaxel arm had an imaging CR at the first imaging timepoint, and however, had progression of disease 5.5 months afterwards.

### GR H-score did not correlate with clinical response

**Fig. 3:**
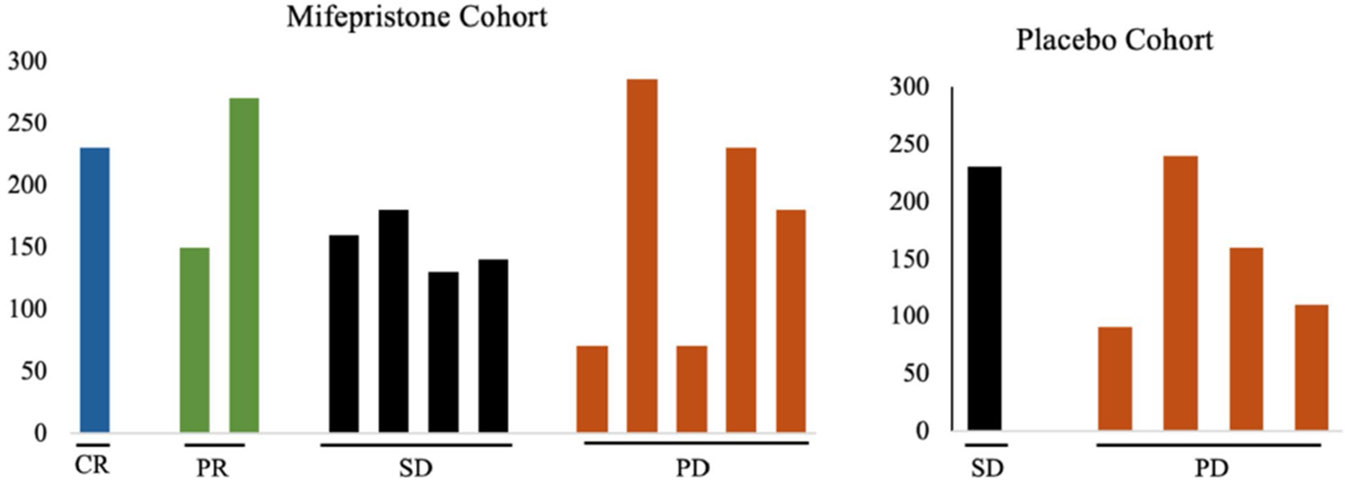
Association of GR expression with clinical responses in patients treated with nab-paclitaxel with or without mifepristone. H-score was used to quantify nuclear GR expression in TNBC tumors. 11 patients in the mifepristone cohort and 5 patients in the placebo cohort had evaluable tissue for GR expression. CR (blue) = complete response, PR (green) = partial response, SD (black) = stable disease, PD (orange) = progressive disease

GR expression (categorized by H-score) was evaluated in both cohorts in archival tissue of primary or metastatic lesion. 17 tumors were available for staining. Within the mifepristone combination arm, the H-score for the one CR tumor was 230, the average H-score in tumors with a PR was 210 (*n* = 2), the average H-score for SD was 152.5 (*n* = 4), and for PD was 167 (*n* = 6) (Fig. 3). While the GR H-scores were numerically higher in the CR and PR groups, this difference is not statistically significant across response categories (*p* = 0.267 in median H-score, *p* = 0.244 in mean H-score) (Table 3).

### Safety and tolerability

Treatment with mifepristone combined with nab-paclitaxel was generally well tolerated, with a safety profile comparable with nab-paclitaxel monotherapy, as summarized in Table 4. The most common treatment-related adverse events (TRAEs) in both groups were fatigue (64%), neuropathy (46%), and neutropenia (24%). The most common grade 3 or greater TRAE was neutropenia. Grade 3 or greater neutropenia occurred more frequently in pts receiving nab-paclitaxel + mifepristone (46% vs 7%). Similarly, febrile neutropenia was also more common in pts receiving combination therapy (23% vs 7%). Serious adverse events were reported in 2 patients; both occurred in the mifepristone combination cohort. One patient had grade 3 dehydration and fatigue and grade 4 electrolyte abnormalities resulting in an inpatient hospitalization. The second patient had grade 3 anemia requiring transfusion. 9 patients (28.5%) required at least one dose reduction of nab-paclitaxel during treatment course; this was due to neutropenia in 7 patients and otherwise related to fatigue, neuropathy, or nausea/vomiting. Dose reductions occurred in 8 patients receiving mifepristone and 1 patient receiving placebo (*p* = 0.004). Despite the frequency of neutropenia, the majority of patients were able to continue treatment with growth factor support. Two patients discontinued study treatment due to AEs. One patient in the placebo arm discontinued due to neutropenia, and one patient in the mifepristone arm discontinued due to nausea/vomiting.

## Discussion

GR activation in ER-negative breast cancer promotes development of chemoresistant phenotypes and is associated with worse clinical outcomes. [8, 12, 19] However, the complexity of the GR signaling pathway and interactions with the tumor microenvironment and immune modulation contribute to an incomplete understanding of a therapeutic role for GR antagonism in advanced ER-negative breast cancer treatment. Taxanes remain an effective and commonly used therapeutic strategy for women with TNBC. However, most tumors are refractory to or develop resistance to taxane-based therapies. Development of innovative strategies to overcome resistance and augment taxane response are desirable. Our study was designed to evaluate the GR antagonist, mifepristone, administered the day before and the day of chemotherapy. This dosing regimen was chosen to reduce drug interactions via inhibition of CYP2C8 by mifepristone which can increase paclitaxel levels. Furthermore, continuous GR antagonism could cause upregulation of compensatory pathways of chemotherapy resistance. Due to the COVID-19 pandemic and the approval of checkpoint inhibitors in advanced TNBC in 2020 [20], this study was terminated early due to poor accrual. In our limited dataset, we did not find that the addition of mifepristone to nab-paclitaxel improved PFS compared to nab-paclitaxel alone. Similarly, ORR was not significantly improved with the addition of mifepristone. There was a trend towards improvement in OS, but this was primarily driven by one long-term responder in the combination group.

As in our prior phase I study, neutropenia was a significant side effect for our patients and seemed to be potentiated by the addition of mifepristone. [15] Pharmacokinetic data in our earlier study revealed a potential drug–drug interaction delaying the plasma clearance of nab-paclitaxel. While many patients in the phase I study developed neutropenia, this was well managed with growth factor support; thus, the 100 mg/m^2^ dose of nab-paclitaxel was chosen for this study. The metabolism of paclitaxel is catalyzed by CYP2C8 and CYP3A4. [21] Mifepristone is an inhibitor of CYP2C8 and CYP3A4. [22] Furthermore, in some patients, it was hypothesized to be due to increased chemotherapy-mediated neutrophil apoptosis in the setting of mifepristone [23, 24]. In the combination arm, development of grade 3 or 4 neutropenia with or without fever was more often seen in patients with stable disease (30%) or progression of disease (40%) compared to PR (20%) or CR (10%) (Table 5). These findings suggest a neutropenic or inflammatory response does not correlate with response to combined GR antagonism and nab-paclitaxel treatment. In most cases, patients were able to continue on treatment with dose delays and usage of growth factor supplementation. One patient who experienced febrile neutropenia during the first cycle was taken off study by the treating investigator due to toxicity. Other toxicities were similar between the two groups.

High expression of GR has been associated with worse outcomes in early-stage TNBC; thus, we explored the association of GR expression with clinical outcomes. H-scores are a semi-quantitative technique to address the heterogeneity of protein staining within tumors. Among our patients with metastatic disease who received mifepristone in combination with nab-paclitaxel, GR protein expression based on H-score was not a predictive biomarker and did not correlate with clinical response. While the median H-score was higher for patients with a CR or PR, this was not statistically significant (Table 3). This analysis was limited by sample size, and H-score data were not available for every patient evaluated in the trial. Moreover, GR nuclear intensity staining differences were seen among tumor slides from the same patient tumor and averaged, demonstrating heterogeneity within TNBC. This is important to address in all studies that aim to identify predictive biomarkers based on protein expression staining. Overall, GR positivity and varying levels of expression were seen in most patients. GR expression as described by H-score may not fully reflect GR signaling pathway activation, and additional markers that reflect GR activation effect may better correlate with clinical outcomes.

There are limitations to this study, primarily the low accrual which precluded us from formally evaluating the study endpoint. While we did not see a benefit from mifepristone in this setting, GR activation in advanced cancers remains prevalent and clinically relevant. The selective GR modulator, relacorilant, has been combined with nab-paclitaxel for the treatment of metastatic solid tumors in phase I trials, including triple-negative breast cancer [16]. In addition, relacorilant in combination with nab-paclitaxel was evaluated in a phase II trial in ovarian cancer yielding increased response rates with the combination [25]. Based on these promising results, a randomized phase III trial of nab-paclitaxel with or without relacorilant in ovarian cancer, ROSELLA, has completed accrual and results are eagerly awaited [26]. These clinical findings in addition to preclinical data that demonstrate GR activation is associated with worse clinical outcomes in ER-negative breast cancer, implicate the need to investigate this pathway in clinical trials and further develop strategies to target chemotherapy resistance in TNBC.

## Figures and Tables

**Table 1:** Baseline Characteristics of Enrolled Patients

Patient Characteristics	Total (*n* = 29)	Nab-paclitaxel + Placebo (*n* = 16 [55.2%])	Nab-paclitaxel + Mifepristone (*n* = 13 [44.8%])
Age, mean (SD) – years	52.8 (11.9)	49.4 (10.1)	56.9 (13.0)
Race/Ethnicity – no. (%)			
Non-Hispanic White	14 (48.2)	9 (56.3)	5 (38.4)
Non-Hispanic Black	11 (37.9)	4 (25.0)	6 (46.2)
Hispanic	1 (3.5)	1 (6.2)	0
Asian/Pacific Islander	1 (3.5)	0	1 (7.7)
Unknown	2 (6.9)	2 (12.5)	1 (7.7)
Number of prior lines of treatment – no. (%)			
0	3 (10.3)	1 (6.2)	2 (15.4)
1	6 (20.8)	3 (18.7)	3 (23.1)
2	5 (17.2)	3 (18.7)	2 (15.4)
Unknown	15 (51.7)	9 (56.4)	6 (46.1)

Abbreviations: SD, standard deviation; no., number

**Table 2:** Clinical outcomes in evaluable patients treated with nab-paclitaxel with or without mifepristone

	Nab-paclitaxel + Placebo	Nab-paclitaxel + Mifepristone
Progression-free survival		
No. of patients evaluated	13	13
Median progression-free survival (95% CI) – months	3.0 (1.0–6.0)	3.0 (1.0–5.0)
*P* value*		0.687
Hazard ratio for disease progression or death (95% CI)		0.87 (0.37–2.01)
Overall survival		
No. of patients evaluated	13	13
Median overall survival (95% CI) – months	6.0 (2.0–15.0)	9.0 (6.0–28.0)
*P* value*		0.325
Hazard ratio for death or last contact (95% CI)		0.67 (0.29–1.16)
Best overall response – no. (%)		
No. of patients evaluated	13	13
Complete response	1 (7.7)	1 (7.7)
Partial response	4 (30.8)	2 (15.4)
Stable disease	1 (7.7)	4 (30.8)
Progressive disease	7 (53.9)	6 (46.2)

Abbreviations: CI, confidence interval; no., number

* *P* value was calculated using the log-rank test

**Table 3:** H-Score Distribution by Response Status in Patients Who Received Nab-Paclitaxel + Mifepristone

Response status					
	CR *n* = 1	PR *n* = 2	SD *n* = 4	PD *n* = 5 (missing *n*=1)	*P* value^a^
H-Score					
Median (minimum – maximum)	230	210 (150 – 270)	150 (130 – 180)	180 (70 – 285)	0.650
Mean (standard deviation)	230	210 (84.9)	152.5 (22.2)	167 (96.0)	0.719
	CR and PR *n* = 3	SD and PD *n* = 9			
Median (minimum – maximum)	230 (150 – 270)	160 (70 – 285)	0.267		
Mean (standard deviation)	216.7 (61.1)	160.6 (69.7)	0.244		

Abbreviations: H-Score, histochemical score; CR, complete response; PR, partial response; PD, progressive disease; SD, stable disease

a *P* value was calculated using ANOVA or Kruskal–Wallis tests between all response groups

**Table 4:** Treatment-Related Adverse Events (TRAE). Grade of TRAE is represented in the nab-paclitaxel with placebo, compared to nab-paclitaxel combined with mifepristone by highest grade per patient. TRAEs experienced by > 1 patient are reported in decreasing frequency

Adverse Event	Grade of AE	Nab-paclitaxel + Placebo (*n* = 15)	Nab-paclitaxel + Mifepristone (*n* = 13)
Fatigue	1	6 (40%)	1 (8%)
	2	4 (27%)	4 (31%)
	3	–	3 (23%)
Neuropathy	1	3 (20%)	7 (54%)
	2	2 (13%)	1 (8%)
Neutropenia	1	–	–
	2	–	–
	3	1 (7%)	5 (38%)
	4	–	1 (8%)
Alopecia	1	2 (13%)	1 (8%)
	2	5 (33%)	5 (38%)
Anorexia	1	–	1 (8%)
	2	–	4 (31%)
Diarrhea	1	1 (7%)	4 (31%)
Dysgeusia	1	1 (7%)	2 (6%)
	2	1 (7%)	4 (31%)
Febrile Neutropenia	3	1 (7%)	3 (23%)
Nail Discoloration	1	–	3 (23%)
Nausea	1	3 (20%)	1 (8%)
	2	1 (7%)	–
	3	–	1 (8%)
Cough	1	–	3 (23%)
Anemia	1	–	–
	2	–	2 (6%)
	3	1 (7%)	2 (6%)
Constipation	1	–	2 (6%)
Headache	1	1 (7%)	2 (6%)
Non-Cardiac Chest Pain	1	–	–
	2	–	2 (6%)
Rash	1	1 (7%)	2 (6%)
	2	1 (7%)	1 (8%)
Transaminitis	3	2 (13%)	1 (8%)

**Table 5:** Severe neutropenia with or without fever associated with clinical response

Grade 3 or greater Neutropenia with or without Fever	Placebo (*n* = 1)	Mifepristone (*n* = 10)
SD	–	3 (30%)
PD	1 (100%)	4 (40%)
PR	–	2 (20%)
CR	–	1 (10%)

Abbreviations: CR, complete response; PR, partial response; PD, progressive disease; SD, stable disease

## Data Availability

No datasets were generated or analysed during the current study.

## References

[1] Matossian M, Chen N, Nanda R (2023) Exploiting therapeutic vulnerabilities in triple-negative breast cancer: successes, challenges, and opportunities. Curr Breast Cancer Rep 15:266–278

[2] Huppert LA, Gumusay O, Rugo HS (2022) Emerging treatment strategies for metastatic triple-negative breast cancer. Ther Adv Med Oncol 14:175883592210869 10.1177/17588359221086916 PMC9003656 35422881

[3] Malhotra MK, Emens LA (2020) The evolving management of metastatic triple negative breast cancer. Semin Oncol 47:229–237 32563561 10.1053/j.seminoncol.2020.05.005

[4] Bianchini G, De Angelis C, Licata L, Gianni L (2022) Treatment landscape of triple-negative breast cancer — expanded options, evolving needs. Nat Rev Clin Oncol 19:91–113 34754128 10.1038/s41571-021-00565-2

[5] De Luca R, Profita G, Cicero G (2019) Nab-paclitaxel in pretreated metastatic breast cancer: evaluation of activity, safety, and quality of life. OncoTargets Ther 12:1621–1627 10.2147/OTT.S191519 PMC6396668 30881017

[6] Blum JL (2007) Phase II study of weekly albumin-bound paclitaxel for patients with metastatic breast cancer heavily pretreated with taxanes. Clin Breast Cancer 7:850–856 18269774 10.3816/CBC.2007.n.049

[7] Conzen SD (2008) Minireview: nuclear receptors and breast cancer. Mol Endocrinol 22:2215–2228 18417735 10.1210/me.2007-0421 PMC2582530

[8] Pan D, Kocherginsky M, Conzen SD (2011) Activation of the glucocorticoid receptor is associated with poor prognosis in estrogen receptor-negative breast cancer. Cancer Res 71:6360–6370 21868756 10.1158/0008-5472.CAN-11-0362 PMC3514452

[9] Williams JB (2009) A model of gene-environment interaction reveals altered mammary gland gene expression and increased tumor growth following social isolation. Cancer Prev Res 2:850–861 10.1158/1940-6207.CAPR-08-0238 PMC4707045 19789294

[10] Wu W (2004) Microarray analysis reveals glucocorticoid-regulated survival genes that are associated with inhibition of apoptosis in breast epithelial cells. Cancer Res 64:1757–1764 14996737 10.1158/0008-5472.can-03-2546

[11] Pang D, Kocherginsky M (2006) Dexamethasone decreases xenograft response to paclitaxel through inhibition of tumor cell apoptosis. Cancer Biol Ther 5:933–940 16775428 10.4161/cbt.5.8.2875

[12] Chen Z (2015) Ligand-dependent genomic function of glucocorticoid receptor in triple-negative breast cancer. Nat Commun 6:8323 26374485 10.1038/ncomms9323 PMC4573460

[13] Wood AJJ, Spitz IM, Bardin CW (1993) Mifepristone (RU 486) – a modulator of progestin and glucocorticoid action. N Engl J Med 329:404–412 8326975 10.1056/NEJM199308053290607

[14] Skor MN (2013) Glucocorticoid receptor antagonism as a novel therapy for triple-negative breast cancer. Clin Cancer Res 19:6163–6172 24016618 10.1158/1078-0432.CCR-12-3826 PMC3860283

[15] Nanda R (2016) A randomized phase I trial of nanoparticle albumin-bound paclitaxel with or without mifepristone for advanced breast cancer. Springerplus 5:947 27386391 10.1186/s40064-016-2457-1 PMC4929099

[16] Munster PN (2022) Overcoming taxane resistance: preclinical and phase 1 studies of relacorilant, a selective glucocorticoid receptor modulator, with nab-paclitaxel in solid tumors. Clin Cancer Res 28:3214–3224 35583817 10.1158/1078-0432.CCR-21-4363 PMC9662918

[17] Eisenhauer EA (2009) New response evaluation criteria in solid tumours: revised RECIST guideline. Eur J Cancer 45:228–247 19097774 10.1016/j.ejca.2008.10.026

[18] Block TS, Murphy TI, Munster PN, Nguyen DP, Lynch FJ (2017) Glucocorticoid receptor expression in 20 solid tumor types using immunohistochemistry assay. Cancer Manag Res 9:65–72 28293120 10.2147/CMAR.S124475 PMC5345989

[19] Bakour N, Moriarty F, Moore G, Robson T, Annett SL (2021) Prognostic significance of glucocorticoid receptor expression in cancer: a systematic review and meta-analysis. Cancers 13:1649 33916028 10.3390/cancers13071649 PMC8037088

[20] Cortes J (2020) Pembrolizumab plus chemotherapy versus placebo plus chemotherapy for previously untreated locally recurrent inoperable or metastatic triple-negative breast cancer (KEYNOTE-355): a randomised, placebo-controlled, double-blind, phase 3 clinical trial. The Lancet 396:1817–1828 10.1016/S0140-6736(20)32531-9 33278935

[21] FDA Label - Abraxane.

[22] FDA Label - Mifepristone.

[23] Vanzulli S (2002) p21, p27 and p53 in estrogen and antiprogestin-induced tumor regression of experimental mouse mammary ductal carcinomas. Carcinogenesis 23:749–758 12016147 10.1093/carcin/23.5.749

[24] Elía A (2023) Beneficial effects of mifepristone treatment in patients with breast cancer selected by the progesterone receptor isoform ratio: results from the MIPRA trial. Clin Cancer Res 29:866–877 36269797 10.1158/1078-0432.CCR-22-2060 PMC9975668

[25] Colombo N (2023) Relacorilant + nab-paclitaxel in patients with recurrent, platinum-resistant ovarian cancer: a three-arm, randomized, controlled open-label phase II study. JCO 41:4779–4789 10.1200/JCO.22.02624 PMC10602497 37364223

[26] Alexander Olawaiye (2022) Rosella: a phase 3 study of relacorilant in combination with nab-paclitaxel versus investigator’s choice in advanced, platinum-resistant, high-grade epithelial ovarian, primary peritoneal, or fallopian-tube cancer. JCO 40:TSP5620–TPS5620

